# Research and Application of Biomass-Based Wood Flame Retardants: A Review

**DOI:** 10.3390/polym15040950

**Published:** 2023-02-15

**Authors:** Yuqing Liang, Hao Jian, Chao Deng, Junxian Xu, Yang Liu, Heejun Park, Mingyu Wen, Yaoxing Sun

**Affiliations:** 1 Department of Wood Material Science and Engineering Key Laboratory, College of Materials Science and Engineering, Beihua University, Jilin 132013, China; 2 Department of Housing Environmental Design, and Research Institute of Human Ecology, College of Human Ecology, Jeonbuk National University, Jeonju 54896, Republic of Korea

**Keywords:** wood materials, intumescent flame retardant, biomass, synergistic flame retardant, environmentally-friendly

## Abstract

Wood is widely used as a construction material due to its many advantages, such as good mechanical properties, low production costs, and renewability. However, its flammability limits its use in construction. To solve the problem of wood flammability, the most common method to improve the fire safety of wood is to modify the wood by deep impregnation or surface coating with flame retardants. Therefore, many researchers have found that environmentally friendly and low-cost biomass materials can be used as a source of green flame retardants. Two aspects of biomass-based intumescent flame retardants are summarized in this paper. On the one hand, biomass is used as one of the three sources or as a flame-retardant synergist in combination with other flame retardants, which are called composite biomass intumescent flame retardants. On the other hand, biomass is used alone as a feedstock to produce all-biomass intumescent flame retardants. In addition, the potential of biomass-based materials as an environmentally friendly and low-cost FR source to produce high-performance biomass-based flame retardants with improved technology was also discussed in detail. The development of biomass-based intumescent flame retardants represents a viable and promising approach for the efficient and environmentally friendly production of biomass-based flame retardants.

## 1. Introduction

**Figure 1:**
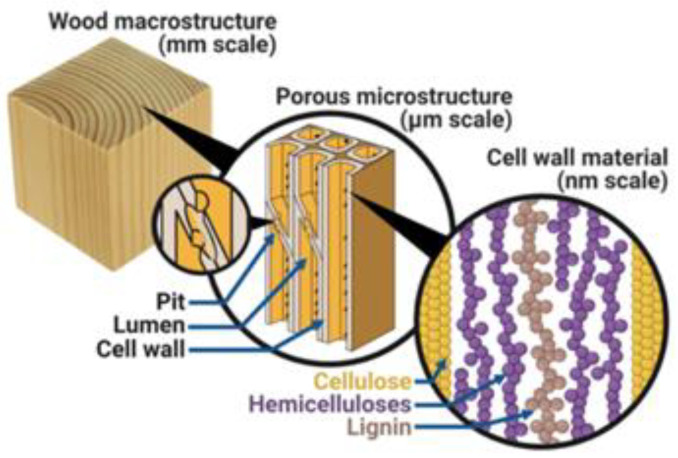
Structure of wood at different length scales. Reproduced with permission [13].

Wood is one of the most widespread and durable natural materials [1]. Due to its low weight, excessive strength, and low price, it is broadly utilized in construction materials, electrical energy consumption, transportation, and furniture [2,3,4]. However, wood is flammable, and its properties limit the use of wood in construction and furniture in densely populated areas [5,6]. In the United States, wooden constructions make up more than 95% of low-rise public structures and more than 50% of industrial buildings [7]. As shown in Figure 1, cellulose, lignin, and hemicellulose are the main components of wood cell walls [8]. The damage caused by fire is a major global problem. Therefore, there is an increasing emphasis on innovative flame-retardant treatment techniques for wood [9]. Studies have shown that adding flame retardants is one of the most effective ways to improve the fire safety of wood materials without affecting their structure and natural appearance [10,11,12].

Since the 1930s, conventional halogen flame retardants have been widely used because of their excellent mass production capacity and low prices [14]. Due to its strong ability to trap free radicals in the gas phase and prevent flame propagation, it exhibits excellent flame retardancy [15,16,17]. The most common halogenated flame retardants are brominated and chlorinated flame retardants, which have been in use since the 1970s [18,19]. These halogenated flame retardants have caused environmental damage due to their toxicity when burned [20]. Table 1 summarizes the advantages and disadvantages of the conventional halogenated flame retardants mentioned above. However, the development of biomass materials has become an important measure for the sustainable development of materials due to the depletion of petrochemical resources, increasing environmental awareness and strict environmental regulations set by governments [21,22]. Based on results reported in the literature, biomass materials were identified as having the potential to intumescent flame retardant [23,24]. Biomass-based materials are new materials made from renewable materials by biological, chemical, and physical means [25]. To improve the flame retardancy of wood while being environmentally friendly, many researchers have attempted to use biomass-based feedstocks to chemically react with flame retardants. One of the simplest, greenest, and most effective ways to protect wood materials from fire is to use intumescent flame retardant (IFR) coatings. These coatings are widely used on wood surfaces [26,27]. Conventional IFR coatings form a porous layer of char residue to protect wood materials and act as a physical barrier to limit the diffusion of combustible gases. Most coatings consist of a combination of film-forming agents and flame retardants (including acid, carbon, and gas sources) [28,29]. Some biomass materials, such as nitrogen [30,31], phosphorus, and sulfur have excellent carbon-forming properties, which can be used in the production and research of intumescent flame retardants [32]. These biomasses employed in flame retardants can be classified into three categories based on their elemental composition: biomass-based charring agents [33], biomass-based acid sources [34], and biomass-based foaming agents [35]. Since charring is an important component of the flame retardant performance of intumescent flame retardants and many biomass-based materials have excellent charring properties, researchers initially focused on lignin, starch, cellulose, chitosan, and other materials with high carbon content and polyhydroxyl groups as charring agents [36]. However, when these biomass materials were added to flame retardants, problems of high addition and poor compatibility occurred. Therefore, there is a need for modification, which will have a greater impact on the use of biomass materials as flame retardants. Natural polyhydroxy materials tend to have poor heat resistance and do not meet the requirements of wood processing, but biomass chemicals have a rich structure and strong designability. Therefore, biomass chemicals such as phytic acid, tannic acid, dopamine, and amino acids as raw materials will have a promising future as flame retardants by giving them properties through structural design. They are excellent at improving the flame retardancy of wood when combined with appropriate chemical reactions. Because of their superior environmental benefits and great flame-retardant efficacy, biomass-based flame retardants have long attracted people’s interest [35,37]. The advantages of biomass and conventional flame retardants are shown in Table 2.

**Figure 2:**
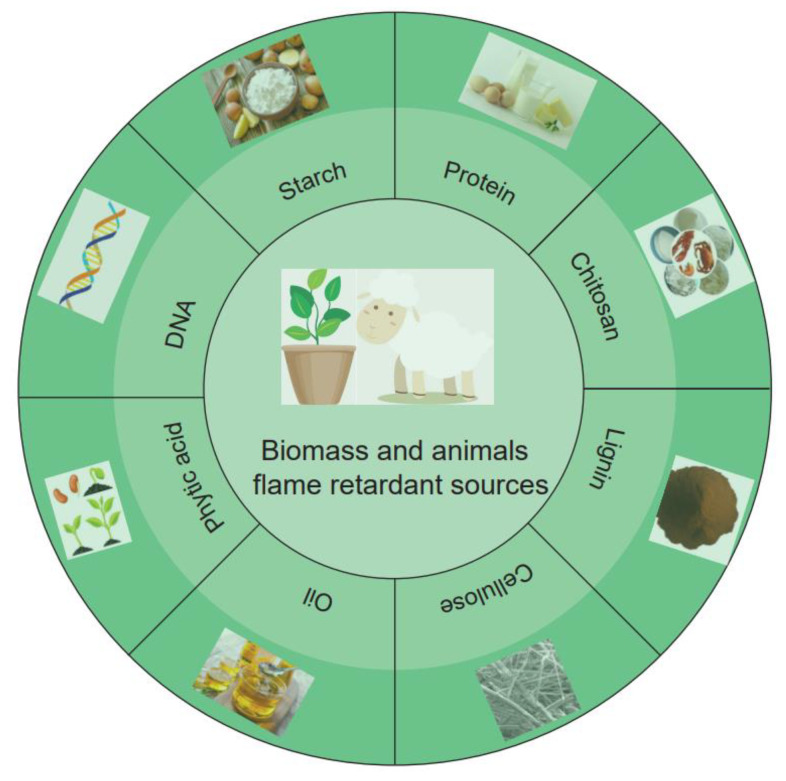
Biomass and animals as biomass-based flame-retardant sources.

There are two common directions of research: one direction is the composite biomass-based intumescent flame retardant obtained using biomass as one of the three sources or as a flame retardant synergist in combination with other flame retardants. The other research direction is only using biomass as feedstock to obtain all-biomass-based intumescent flame retardants. This paper classifies plants and animals as sources of biomass-based flame retardants, reviews the current status of research on the two major types of biomass-based intumescent flame retardants in the wood field, and describes the many challenges faced in their development. Figure 2 summarizes the sources of biomass flame retardants from biomass and animals.

## 2. Composite Biological Intumescent Flame Retardant

### 2.1. Biomass-Based Carbon Source

Most biomass materials have a multi-hydroxyl chemical structure, which can carry out different chemical reactions and can be heated to form char [39]. As such, they have the potential to become a source of carbon for flame retardants that can not only replace polymeric materials in the future but can also help to conserve non-renewable resources and ensure the sustainable development of humanity [40,41]. The carbon source is a carbon-forming agent with multi-carbon and a multi-functional group structure [42,43]. In the process of combustion, a porous carbon layer will be formed under the action of an acid source to achieve protection. Conventional carbon-forming agents are represented by pentaerythritol, dipentaerythritol, and other small molecules. However, small molecule carbon-producing agents are not appropriate for sustainable development due to their low flammability and possible limited petroleum resources [44,45]. It was found that natural biomass macromolecules with multiple carbon and hydroxyl groups from nature, such as natural starch [46,47], sugars [48], lignin [49], and cellulose [50], also have good carbon-forming properties and can be used as biomass-based carbon sources to replace traditional petroleum-based carbon sources.

Lignocellulose is one of the most popular thermal and acoustic insulation materials made from renewable materials [51,52]. Gebke et al. [53] modified wheat starch. They used a typical phosphate/urea reaction method as a flame-retardant additive to wood cellulose for the modification. The results demonstrated that starch-modified flame retardants are capable of matching the fire resistance of industrial flame retardants currently employed in the wood cellulose market. As a result of the functionalization, fireproof phosphates up to 38 wt.% and nitrogen groups up to 8.3 wt.% were added. When soluble additives were used in combustion experiments, the lowest amounts of burning were recorded at a phosphate content of 3.5 wt.%. When compared to wood cellulose that has not been treated, smoldering effects may be greatly reduced. The results of the experiment demonstrate that starch-based flame retardants are fundamentally applicable to processing wood.

Baishya et al. [54] used starch and coniferous wood as raw materials and water as a solvent to prepare biomass-based nanocomposites. To prepare wood starch nanocomposites, wood starch nanocomposites (WSNC) were prepared using methyl methacrylate (MMA)-modified starch, dimethyl dihydroxyethylurea (DMDHEU), wood flour, glycerol, and montmorillonite (MMT) as raw materials. The montmorillonite-modified starch composites were prepared with three different concentrations of montmorillonite, and the flame retardancy of the montmorillonite-modified starch composites was evaluated by limiting oxygen index test and thermogravimetric analysis. The experimental results showed that the T_i_, T_m_, and T_D_ values of the biomass-based nanocomposites were higher than those of a single wood, indicating improved thermal stability. This study shows that this environmentally friendly manufacturing process can significantly improve the flame retardancy of wood, which can be used in various fields.

Lignin is one of the main components of lignocellulosic biomass, along with cellulose and hemicellulose [55,56]. In plants, lignin bridges the gap between cellulose and hemicellulose, keeps the lignocellulose matrix together, and functions as an adhesive to provide cell wall strength and rigidity. Herbaceous plants, hardwood, and softwood typically contain 15 to 20%, 20–25%, and 20–30% percent lignin, respectively [57,58]. Wei et al. [59] studied the preparation of a fully degradable flame retardant wood/poly(lactic acid) (PLA) biocomposite (FPW) with low cost and excellent mechanical properties. A series of lignin-based flame retardants (LMDs) with different lignin contents were first synthesized. Then, FPW was made from melt-blended PLA, wood powder, poly(butyleneadipate-co-terephthalate), triglycidyl isocyanurate (TGIC), and LMD. To further improve the mechanical properties of FPW, TGIC was added as a reactive coupling agent. Meanwhile, nitrogen in TGIC and phosphorus in DOPO play a synergistic role in the combustion process of FPW composites, improving the flame retardancy of FPW composites. This study shows that lignin plays a key role in the flame retardancy of prepared PLA biocomposites. Flame-retardant PLA biocomposites also have excellent biodegradability and can be more widely used.

**Figure 3:**
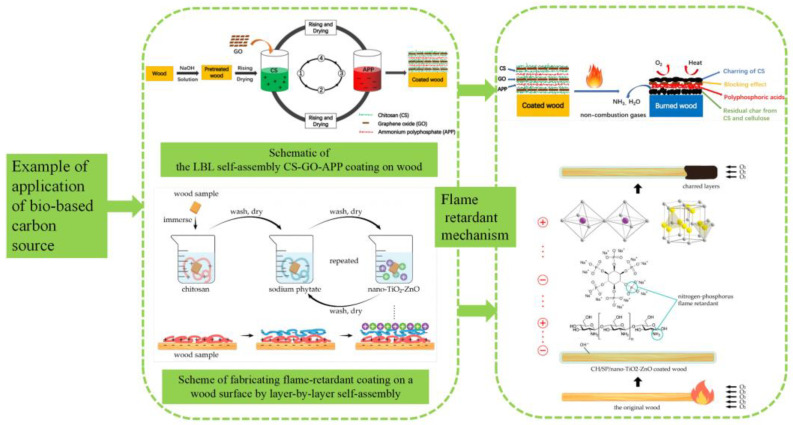
Application of biomass carbon sources [60,62].

Yan et al. [60] used a ternary flame retardant system of chitosan (CS), graphene oxide (GO), and ammonium polyphosphate (APP) to prepare efficient and durable flame retardant coatings on wood surfaces using a layer-by-layer self-assembly method. Figure 3 summarizes the preparation of flame-retardant coatings. Thermogravimetric analysis revealed that the CS-GO-APP coating greatly increased the char residue while dramatically lowering the beginning and maximum thermal breakdown temperatures of the wood. Due to the early degradation of CS and APP, which enhanced the thermal stability of the modified wood. Limiting oxygen index and cone calorimetric analysis of the original and coated woods after modification with CS-GO-APP showed that the fire safety of the modified wood was improved. SEM images of char residue after combustion proved that the combined CS-GO-APP coating promoted the formation of a char residue layer on the wood surface, blocking the heat and flame spread, thus protecting the wood from the fire. The surface char density of wood with a flame-retardant coating is higher than virgin wood. This is because the polyphosphate generated by APP degradation catalyzes cellulose charring as well as the promotion of polyhydroxy CS during combustion, which prevents flame propagation. The excellent fire safety of the modified wood is due to the key synergistic effect of CS. Meanwhile, the self-assembly method in this study has the potential to reduce the fire risk of wood products.

Uddin et al. [61] combined chitosan (CS), nanocellulose, and boric acid (BA) in a transparent hybrid film. The results showed that the use of chitosan and boric acid together resulted in enhanced flame retardancy. However, they became self-extinguishing when combined with 30 wt.% of boric acid. Chitosan was added to cellulose to observe the synergistic flame-retardant effect. In the absence of boric acid, chitosan cellulose nanofiber (CS-CNF) films exhibited self-extinguishing and flame-retardant properties, indicating the advantage of adding chitosan. Moreover, the addition of boric acid enhanced the thermal stability of CS, CNF, and CS-CNF films, and improved carbon formation. It was shown that chitosan and boric acid exhibited excellent synergistic flame-retardant effects.

Zhou et al. [62] investigated chitosan/sodium phytate/TiO_2_-ZnO nanoparticle composite coatings (CH/SP/Nano-TiO_2_-ZnO), which were applied to wood surfaces by layer-by-layer self-assembly. The effective combination effect between the individual components of the coating gave rise to the excellent flame-retardant properties of the coating. The experimental results show that the flame-retardant coating has an effective intumescent flame-retardant system and physical barrier. This experiment shows that chitosan can also exhibit excellent synergistic flame retardancy with metal oxides. The flame-retardant mechanism of CH/SP/Nano-TiO_2_-ZnO composite films is summarized in Figure 3.

Lignin with a phenolic structure can be used as a flame retardant. The thermal properties of lignin, such as thermal degradation, molecular weight, and lignin purity, affect the performance of lignin as a flame retardant through the formation of significant char residues [63,64]. However, lignin faces significant limitations in meeting industrial requirements in the final polymer. To improve the flame-retardant properties of lignin, researchers have modified lignin with nitrogen and phosphorus chemicals and shrunk lignin to the nanoscale to reduce the aggregation of lignin with wood materials. This has resulted in excellent flame-retardant properties [65]. Currently, research concerning biomass-based carbon sources tends to be diversified. Researchers are using natural products such as starch, sugar, and lignin as biomass-based carbon sources in combination with other flame retardants such as ammonium poly-phosphate to produce biomass-based intumescent flame retardants. Such biomass-based carbon sources have a significantly better flame-retardant effect than traditional petroleum-based carbon sources, such as pentaerythritol. They are natural renewable resources; it can alleviate the oil shortage crisis and meet current demand [66,67,68].

They also meet the trend of developing green environmental protection [69]. However, most biomass-based carbon sources have not yet overcome the drawback of the need for a large number of intumescent flame retardants. In addition, they cannot yet eliminate the detrimental effects of flame retardants on the mechanical properties of wood materials [70]. To address the above issues, many researchers have attempted to incorporate silicon-containing compounds into biomass char sources to improve the thermal stability of biomass char sources and increase their compatibility with wood substrates, and preliminary results have been obtained [66,68]. Under the premise of ensuring the flame-retardant effect, effectively reducing the amount of biomass-based carbon source and improving its thermal stability is the future development direction. Therefore, the future development trend of biomass-based flame-retardant carbon formers is focused on the following aspects:

1. To further improve the flame retardancy and char yield of biomass-based flame retardants, the grafting rate of biomass-based char forming agents must be improved through molecular design and compounding with metal ions such as nitrogen and silicon.
2. Most polysaccharides degrade at temperatures above 200 °C and undergo discoloration. This affects the appearance of flame-retardant materials. The thermal stability of flame retardants based on biomass can be improved by chemical modification or by cross-linking.
3. The physical properties of biomass-based flame retardants, such as water resistance, are improved by chemical modification or surface treatment.

### 2.2. Biomass-Based Acid Source

Due to the complexity of biomass materials, the use of biomass-based materials as a flame-retardant synergist with combustion-supporting chemicals such as ammonium polyphosphate is the focus of most research on biomass-based intumescent flame retardants [41,71,72]. Researchers have also used biomass to create polyelectrolytes as biomass-based intumescent flame retardants to reduce the flammability of wood and cotton fabrics [73,74,75].

A myo-inositol ring and six symmetrically linked phosphate groups make up PA, also known as myo-inositol hexaphosphate (IP6) [76,77]. In many plants, including beans, cereal grains, and oilseeds, phytic acid can be found as a renewable and environmentally-friendly organic acid source [78,79,80]. Due to its low cost, high phosphorus content, and sustainability, it is mainly used as an inorganic flame retardant in cotton fabrics [81,82], wood materials, etc. In a recent study on intumescent flame retardant, it was found that the best natural acid source for producing intumescent flame retardant is phytic acid [83], a biomass acid source with up to 28% rich phosphorus.

**Figure 4:**
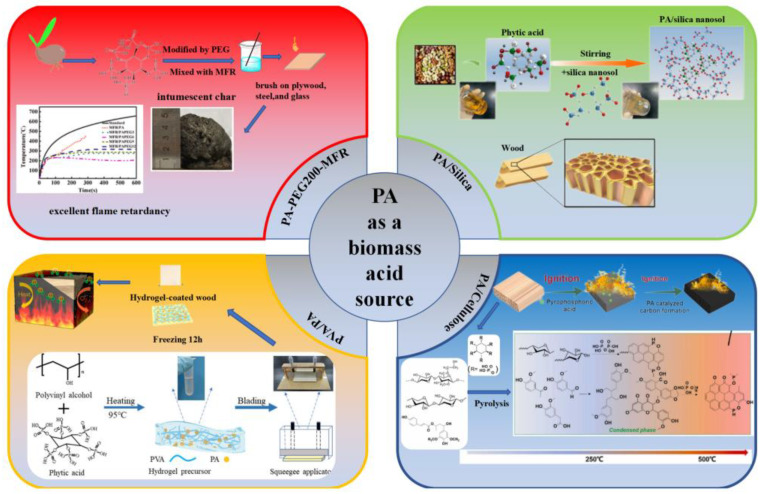
Flame retardants use phytic acid as a biomass acid source [84,85,87,88].

Most of the three sources of intumescent flame retardants are inorganic acids or dehydrating agents that can generate strong acids in the combustion process [33]. Traditional acid sources include phosphoric acid, ammonium polyphosphate, phosphate ester, etc., which are non-renewable chemical raw materials. To solve the growing energy shortage, researchers are trying to develop biomass-based acid sources. Natural phosphorus-containing substances such as phytic acid have been successfully prepared as biomass-based acid sources [83]. The practical application of phytic acid as a biomass acid source is summarized in Figure 4.

Tian et al. [84] prepared phytic acid-based phosphate esters (PAPEGs) by reacting phytic acid with polyethylene glycol 200 (PEG-200) in different ratios. PAPEGs and melamine formaldehyde resin (MFR) were thermally cured to obtain intumescent flame-retardant coatings with different ratios. MFR/PAPEG coatings provide excellent flame retardance and water resistance. This is due to the generation of phosphoric acid in the coating, which dehydrates and carbonizes the PEG, resulting in the formation of graphitized carbon. At the same time, the nitrogen-containing intermediates simultaneously release non-combustible gases that swell the char and interrupt the combustion reaction. Phytic acid has also been shown to have an important role in the inhibition of combustion.

Chen et al. [85] constructed a phytic acid (PA)-silica hybrid system in wood by vacuum pressure impregnation to improve its flame retardancy and smoke suppression. The improvement in the flame-retardant and smoke-suppressing properties of wood is that the char residues form a completely coherent and cross-linked structure. This can prevent further diffusion of flammable gases and smoke during combustion [86]. Phosphorus in PA can catalyze the dehydration of cellulose in wood and promote the formation of charcoal at the initial stage of pyrolysis. Due to its environmental protection properties, the PA-silica system shows great application potential in the field of fire protection. This process can be extended to make other phosphorus and silicon materials to improve the fire safety of the wood.

To enhance the flame-retardant properties of wood through a freeze-thaw cycle, Zhao et al. [87] developed a polyvinyl alcohol (PVA)/phytic acid (PA) hydrogel coating that is easy to process, environmentally-friendly, and efficient. Compared with pure PVA hydrogel coating, PVA/PA hydrogel coating has better adhesion and fire safety performance. When burned, hydrogel-coated wood shows good fire retardancy because the water in the coating is heated and evaporates first, absorbing most of the heat and reducing the surface temperature of the wood. As a phosphorus-based flame retardant, PA promotes the dehydration of wood into carbon and prolongs the high-temperature combustion process of wood. This results in the formation of a denser and thicker layer of carbon on the surface of the wood, inhibiting external heat and oxygen transfer. The multiple fire protection mechanism of the hydrogel coating enables wood to meet fire protection requirements as a structural material and ensures its wide range of applications. The study shows that phytic acid can be used not only in flame retardant coatings, but also in hydrogels as well. It also demonstrates that phytic acid, which is an easily accessible and environmentally friendly biomass material, has a wide range of potential applications.

Wang et al. [88] introduced biomass-based acid sources into the cellulose lumen to enhance the flame retardancy of wood. The limiting oxygen index value reaches 37.2% and the wood sample is difficult ignite. The treated wood samples show not only excellent flame-retardant properties, but also excellent mechanical properties. This is due to the hydrogen bonding between the phytic acid molecules and the cellulose. This study provides a feasible way to obtain flame-retardant wood.

By in-depth research on the literature, it is found that there are few kinds of studies on biomass-based acid sources, mainly focusing on biomass phytic acid with a high phosphorus content. By introducing nitrogen-containing and carbon-containing flame retardants to the phytic acid, biological intumescent flame retardants with excellent flame retardancy can be prepared. However, the extraction and processing of phytic acid are complicated and the price is high, which is not conducive to the development of industrialization. Therefore, it is far from enough to only study phytic acid as a biomass-based acid source. Other natural polyphosphoric biomasses, such as protein, can be explored as a potential biomass-based acid source [89]. With the deepening of the study of biomass and the continuous progress of extraction technology, it is bound to develop new biological acid sources with reasonable prices and excellent effects in the future.

### 2.3. Biomass-Based Gas Source

The gas source of the three sources of intumescent flame retardant is also known as a blowing agent and is composed of nitrogen-containing compounds [90]. The conventional gas sources are melamine, urea, etc., and are also difficult to regenerate chemical raw materials. Therefore, scholars began to try to use protein, pyrimidine, etc., as biomass-based gas sources to prepare biomass-based intumescent flame retardants.

Weng et al. [91] synthesized and tested a durable protein-based flame retardant by fusing an adhesion domain from mussel foot protein-5 (mfp-5) or a cellulose-binding domain (CBD) with flame retardant proteins (SR protein and alpha casein). In the wood flame retardant test, the flame retardancy of SR protein and α-casein flame retardant protein with CBD adhesion domain increased by 50.0% and 43.3%, respectively. In addition, it was found for the first time in the combustion test that amino acids also exhibit certain flame retardancy. The building blocks of proteins are amino acids, and nitrogen has also been found to contribute to the flame resistance of proteins. Overall, flame-retardant proteins with adhesion domains show great promise as green biomass flame retardants.

To prepare wood/PA/uracil fire retardant wood from natural wood by physical infiltration into aqueous solutions, Zhang et al. [9] used phytic acid extracted from plant seeds and uracil extracted from ribonucleic acid as aqueous solutions. The prepared solution is durable, fire-resistant, and environmentally friendly. Compared with natural wood, the peak heat release rate, total heat release rate, and smoke production rate of flame-retardant treated wood decreased significantly. FR wood can achieve a 31.8% limit oxygen index value and UL-94 V-0 rating.

Scholars have used nitrogen-based biomass as a biomass-based gas source to obtain biomass-based intumescent flame retardants that work synergistically with existing acid and carbon sources. Since nitrogen-containing biomass can release non-flammable gas in time during combustion, such biomass-based intumescent flame retardants have remarkable flame-retardant effects. In contrast to the non-renewable raw materials and complex production techniques involved in the preparation of melamine, protein and pyrimidine are derived from renewable biomass and can also be biodegradable, making the preparation process relatively simple [92].

### 2.4. Other Biomass-Based Flame Retardant Synergists

Typical commercial intumescent flame-retardant systems are generally composed of ammonium polyphosphate (APP) and pentaerythritol (PER), where APP is an acid source and an air source, and PER is a carbon source [93,94]. It is often used as a flame retardant by coating wood and other materials [95,96]. However, the flame retardancy efficiency of this intumescent flame-retardant system is relatively low. In addition, flame-retardant synergists are usually required to achieve satisfactory results. To achieve the goal of green and sustainable development and high flame-retardant efficiency, many scholars have tried to prepare biomass-based flame-retardant synergists and achieved a better flame-retardant effect.

**Figure 5:**
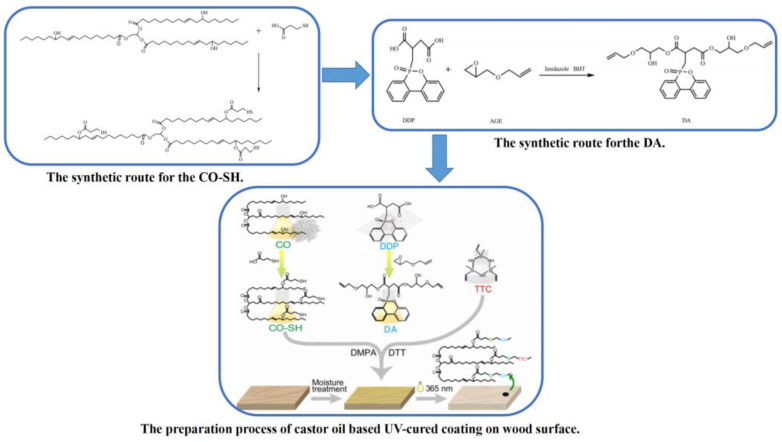
The preparation process of castor oil-based UV-cured coating on a wood surface. Reproduced from [97].

Wang et al. [97] prepared a multifunctional bio-mercaptan from castor oil. Figure 5 summarizes the preparation process of castor oil-based UV-curable paint for wood surfaces. The castor oil-based UV-curable flame-retardant coating containing P/Si/S was successfully prepared on the wood surface by thiol-ene click reaction. On the one hand, the non-combustible gases SO_X_ and CO_2_ produced by S and C in the coating dilute the oxygen concentration. The phosphorus-containing compounds produced by the decomposition of the coating also consume oxygen due to the synergistic effect of P and S. On the other hand, the polyphosphate produced by flame-retardant degradation decomposes the organic matter in the coating. As a result, the coating forms a more continuous and dense carbon layer containing Si and P on the wood surface. These barrier coals inhibit heat and oxygen transfer and prevent the wood substrate from burning completely after ignition. The experimental results show that bio-mercaptan is an environmentally friendly flame retardant.

Huang et al. [98] prepared a novel polybasic carboxylic acid (HCPVC) by nucleophilic substitution reaction using vanillin and hexachlorocyclotriphosphazene as raw materials. HCPVC was used as a curing agent in wood epoxy coatings. The wood sample has excellent UL-94 V-0 flame retardancy and a limiting oxygen index value of 30.7%, which is much higher than that of ordinary epoxy coatings and uncoated wood samples. In the combustion experiment, it was found that the coating formed a dense char residue, which prevented the effective transfer of heat and combustible gas or volatiles to the condensed phase. At the same time, the flame-retardant coating produces more inert gases and other non-flammable volatile products, which act as diluting agents and promote the formation of an intumescent carbon layer. This experiment shows that vanillin as a biomass material is also one of the candidates for future green flame retardants.

Ozkan et al. [99] treated Anatolian black pine with aqueous solutions of 10%, 20%, and 30% flame retardants consisting of di-ammonium phosphate (DAP), borax, boric acid, and glucose. The heat treatment of the treated wood reduced water uptake and improved stability and mechanical properties. The flame-retardant treated wood reduces water intake, improves dimensional stability, and also improves mechanical and thermal properties. At the same time, DAP/glucose promotes the fixation of phosphorus, which makes the wood have long-term fire resistance. For this experimental result, it is shown that glucose can be used as a reliable biomass material for a wide range of fire-retardant applications.

Currently, existing biomass-based effectors mainly focus on enhancing the flame retardancy of polyphosphate and pentaerythritol flame retardant systems. The biomass that has synergistic effects includes nucleotides and nitrogenous bases, etc. This is mainly achieved by promoting APP and PER flame retardant systems to produce a more stable and complete carbon layer protection layer to improve the flame-retardant performance of composite materials. This has great reference value for the production and application of APP and PER flame retardants. However, this kind of biomass also has the disadvantage of high price due to complex separation and other reasons. Therefore, in future research and development, not only sustainable development but also economic cost should be taken into account to develop practical biomass-based flame-retardant synergists.

## 3. All-Biomass Intumescent Flame Retardant

Although the above biomass-based intumescent flame retardants have been successfully prepared, their flame-retardant effect is remarkable. However, a large number of organic solvents are usually used in the preparation or treatment of biomass-based flame retardants, and most of the materials come from non-renewable petroleum feedstocks [100]. Therefore, it is still necessary to develop fully biomass-based, sustainable, and truly green intumescent flame retardants. A small number of scholars are currently working on all-biomass intumescent flame retardants.

Li et al. [101] enhanced the flame retardancy of wood using whole-cell vacuum pressurized impregnation and a water-soluble flame retardant consisting of phytic acid (PA), hydrolyzed collagen (HC), and glycerol (GL). The results showed that the flame retardants impregnated the wood and gave it good flame-retardant properties. The flame-retardant impregnated wood showed good charring formation performance and high graphitization. After burning, the dense carbon layer releases non-flammable volatile gases, such as H_2_O, CO_2_, and NH_3_, to prevent further combustion of wood. Therefore, flame retardants synthesized using biomass materials such as phytic acid and hydrolyzed collagen have proven to be effective fire retardants for wood.

Leng et al. [102] used a green approach to design and synthesize a fully biomass-based flame retardant, phytanic acid-tyramine salt (PATA for short), using deionized water as the reaction solvent. Then PATA was combined with ammonium polyphosphate (APP) to synergistically improve the flame-retardant properties of wood plastic composites (WPC). The synergistic system has good flame retardancy, heat reduction, and smoke suppression properties. The synergistic flame-retardant system has good thermal stability and smoke suppression performance. Through the analysis of the flame-retardant mechanism, it is found that PATA can promote the formation of carbon in the combustion process, thus having a synergistic flame-retardant ability in the presence of APP. The PATA/APP synergistic system simultaneously creates a stable char layer during combustion that contains P-N-C and P-O-C structures, which is one of the factors contributing to the material’s remarkable flame retardancy. This experiment offers a strategy to increase the flame retardancy of wood-plastic products that are green and environmentally favorable overall.

Research shows that although the development of all-biomass intumescent flame retardant started late, its potential is huge. At present, there are mainly two kinds of all-biomass intumescent flame retardants: one is natural phosphoric acid complex, which can become all-biomass intumescent flame retardants without complex treatment, and the other is to chemically react two or more kinds of biomass to prepare all-biomass intumescent flame retardant, both of which have a good flame-retardant effect.

## 4. Challenges and Future Perspective

Many renewable biomass resources have undergone extensive fire resistance testing to produce green, environmentally friendly biomass-based intumescent flame retardants. The results show that certain raw materials have the potential to be developed into biomass-based flame retardants. However, the development of biomass-based intumescent flame retardants currently faces many challenges:

1. The amount of biomass-based intumescent flame retardants is still very high, which has a significant negative impact on the mechanical properties of these materials.
2. Although biomass materials such as chitosan and phytic acid are naturally obtained, they require a number of complex extraction and processing steps during the production process. This inevitably leads to a significant increase in their cost and severely limits their industrialization. For example, Wang et al. [88] introduced biomass-based acid sources into the cellulose lumen. One of the complex steps is that it needs to first delignify the wood and then adsorb the phytic acid into the wood container to enhance the flame retardancy of the wood.
3. Because the structural properties of most biomass materials are still being explored, most researchers use them as char-forming agents in the current development of biomass-based intumescent flame retardants and flame-retardant synergists with mature acid sources such as ammonium polyphosphate. However, research on biomass acid sources, gas sources, flame retardant synergists, and all-biomass intumescent flame retardants is scarce. For example, Leng et al. [102] used phytic acid tyrosine salt (PATA) to react with deionized water. Then, PATA and ammonium polyphosphate were mixed to effectively increase the flame resistance of wood plastic composites.
4. The poor heat resistance of biomass materials has led to problems such as degradation and discoloration during heat treatment.

To meet the above challenges, the future development trends of biomass-based intumescent flame retardants are as follows:

1. The sources of biomass-based intumescent flame retardants should also be expanded from multiple directions. For example, focusing on the comprehensive utilization of biomass waste resources, deepening biomass research, and continuously improving extraction technology to find more novel and diverse biomass with different structures.
2. Through functional modification of a large amount of biomass and the introduction of multifunctional functional groups such as triazine structures and silicon-containing substances, the heat resistance of biomass fire retardants can be improved. Its compatibility with the substrate can be enhanced, and a more stable and continuous protective layer of carbon can be formed. This can not only improve the flame-retardant effect of biomass materials, but also reduce the adverse effect on the mechanical properties of the wood substrate and solve the degradation of biomass at high temperatures and discoloration. Although the development of biomass-based intumescent flame retardants is currently facing great challenges, it is still an essential part of achieving sustainable development. It is believed that with the progress of science and technology, highly efficient, green, and environmentally friendly biomass-based intumescent flame retardants will be obtained.

## 5. Conclusions

As the application of wood materials becomes more extensive, the requirements for the flame-retardant market are becoming more stringent. The development of flame retardants for wood materials has a long history, ranging from halogenated flame retardants to nitrogen-phosphorus flame retardants, and finally to the intumescent flame retardants that have emerged in recent years. Phosphorus flame retardants are more complicated, and their flame-retardant method covers both gas-phase and condensed-phase flame retardants. However, phosphorus flame retardants have some disadvantages in use, such as high moisture absorption and poor compatibility with wood materials. As a very environmentally friendly flame retardant, nitrogen-based flame retardants have the advantages of low toxicity, smoke suppression, low corrosion, and excellent thermal stability. However, the use of nitrogen-based flame retardants alone often does not achieve the required flame-retardant effect, which requires the addition of other types of flame retardants used in combination. Thus, biomass-based intumescent flame retardants will eventually occupy the center of the flame-retardant system. The green and recyclable advantages of biomass-based flame retardants, such as degradability and non-toxicity, will become the hot spot of flame-retardant research in the future due to current environmental and resource recycling requirements. However, from the current point of view, the research on biomass-based flame-retardant systems is still in the introductory stage, but it has significant research significance. In addition, a single flame retardant will have an impact on other properties of the material, and the compounding of biomass materials with flame retardants or the synthesis of P, N, and S multi-element flame retardants will become a hot spot for future flame-retardant development.

## Figures and Tables

**Table 1:** Advantages and limitations of conventional flame retardants.

Category	Halogen	Non-Halogen		
Type	Brominated Chlorinated	Phosphorus-based	Nitrogen-based	Metallic hydroxide
Reaction mechanism	Releases gaseous hydrogen halide into the air, isolating oxygen and interacting with active free radicals OH• and H•	Active phosphorus-containing radicals such as PO• and PO_2_• act in the gaseous phase, reacting with free-radical OH•	Mostly melamine based. Condensed-phase melamine transforms into cross-linked structures, producing solid-phase char layers	Acts as a catalyst to produce carbonized residues during combustion
Advantages	Low-cost, high flame-retardant efficiency and wide applicability	High efficiency, less influence on light stability, and hinder reignition	Low volatility, halogen-free, low toxicity, no corrosive gas	Non-toxic, good stability, no toxic gas produced at high temperature, low price, a wide range of sources
Disadvantages	Produces large quantities of smoke and toxic, corrosive gases when burned	High volatility, poor heat resistance, and unsatisfactory compatibility	Poor compatibility, easy-to-absorb moisture, and affects the physical performance of the substrate	Poor acid resistance and susceptibility to acid

**Table 2:** Advantages of biomass and conventional flame retardants.

Advantages of Bio- FRs	Advantages of Conventional FR
No toxic gases were released during the combustion	Long usage period
Self-degradable	Non-biodegradable
Similar to traditional flame retardants	Excellent mechanical and flame-retardant properties
Low cost of production	Cost-effective and convenient preparation

Note: sources from ref. [38].

## Data Availability

Not applicable.
